# Implementation of physical activity interventions for people with inflammatory arthritis: an overview and future recommendations

**DOI:** 10.1093/rap/rkac094

**Published:** 2023-01-24

**Authors:** Nicola Cornwall, Laura Swaithes, Charlotte Woodcock, Emma L Healey, Samantha L Hider

**Affiliations:** School of Medicine, Keele University, Keele, UK; School of Medicine, Keele University, Keele, UK; School of Medicine, Keele University, Keele, UK; School of Medicine, Keele University, Keele, UK; School of Medicine, Keele University, Keele, UK; Rheumatology Department, Haywood Hospital, Midlands Partnership Foundation Trust, Staffordshire, UK

**Keywords:** physical activity, implementation, inflammatory arthritis, osteoarthritis

## Abstract

Regular physical activity is important for both physical and mental health. This is particularly important for people with inflammatory arthritis, because of the benefits on both disease-specific and systemic outcomes and the increased risk of comorbidities such as cardiovascular disease. Despite a wealth of evidence supporting physical activity interventions, there remains a significant gap in implementation into routine care. This overview describes what implementation is, examines why it is important to consider implementation approaches to improve uptake of physical activity, highlights factors that influence successful implementation using exemplars from both osteoarthritis and inflammatory arthritis and recommends where future research is needed.

Key messages

- Regular physical activity is important for physical and mental health, but uptake is poor.
- Delays exist in translating interventions to improve physical activity into routine clinical practice.
- Implementation may be influenced at the individual (professionals and the public), organizational and policy levels.
- Considering implementation strategies in the design of physical activity interventions increases the likelihood of an intervention being widely adopted.

## Introduction

The importance of engaging in physical activity to benefit physical and mental health is well documented [1] and is a key target for public health action. The 2020 World Health Organization (WHO) guidelines [2] reaffirm that all adults should regularly undertake physical activity and that some physical activity is better than none. These guidelines also highlight the benefit of increased physical activity for people living with disability and chronic conditions. Although inflammatory arthritis (IA) was not specifically considered by the WHO, recommendations from international groups (e.g. EULAR) highlight the importance of physical activity in IA management based on proven effectiveness, feasibility and safety [3], advocating that advice around physical activity be an integral part of self-management and standard patient care [4]. There is increasing evidence that physical activity is important for improving both disease-related (fatigue and disability) and systemic outcomes (such as cardiovascular disease risk) for people with RA [5] and thus should be a key target for intervention in people with IA.

Globally, one in four (27.5%) adults do not meet the recommendations for physical activity [2], and these rates are likely to be even lower in older people, those with disabilities or those living in areas of socio-economic deprivation [6]. Patients with ongoing pain demonstrate reduced levels of daily physical activity [7], and physical inactivity is not only a risk factor for health, but also a possible risk factor for chronic pain [8]. The Quantitative Patient Questionnaires in Standard Monitoring of Patients with Rheumatoid Arthritis (QUEST-RA) study across 21 countries highlights that only 13.8% of people with RA performed physical activity at recommended levels [9].

Thus, despite the wealth of evidence supporting physical activity, uptake of these recommendations is lacking and further work to implement physical activity recommendations into clinical practice for people with IA is needed. This overview describes what implementation is, examines why it is important to consider implementation approaches to improve the uptake of physical activity by patients, highlights factors that influence successful implementation using exemplars from both OA and IA and recommends where future research is needed.

## What is implementation and how can this be achieved?

Implementation is defined as the process of ‘active and planned efforts to mainstream an innovation within an organization’ [10]. Research has consistently shown that uptake of many evidence-based interventions is limited, significant delays exist in translating research findings into clinical practice and approximately one-third of patients do not receive care according to the best scientific evidence [11, 12]. Successful implementation of research evidence is therefore central to ensuring that high-quality, evidence-based care is delivered to patients.

The interest in implementation-focused research is supported by the updated Medical Research Council guidance for evaluating complex interventions [13], with recommendations for early consideration and continued use of implementation questions throughout the phases of intervention development, feasibility testing and process and outcome evaluation, in order to increase the likelihood of a developed intervention being widely adopted and embedded.

Despite this, successful implementation is notoriously challenging due to the multifaceted nature of complex interventions that may require different implementation strategies in different settings. Context is a key consideration for implementation science [14] and, as such, there is no ‘one-size-fits-all’ approach. However, it is important to acknowledge and understand the real-world context in which an intervention is to be implemented to begin to identify and address potential barriers. The use of theory is advocated to assist with planning, understanding and evaluating the factors that influence the effectiveness of implementation [15]. Furthermore, collaborative approaches (such as co-production) that engage key stakeholders, including patients, and work across professional and organization boundaries can ensure the research and implementation plans are appropriate for end-users and hence more likely to be adopted in practice [16].

## Implementation approaches for physical activity interventions

Despite a significant increase in the number and effectiveness of interventions promoting physical activity in the general adult population [17], only 3% of such interventions have been implemented on a large scale over the past 3 decades [18], highlighting a significant research–practice gap [19].

Recent work has focused on exploring the use of implementation science in physical activity interventions. A Delphi study identified 37 factors important for both general and specific ‘introduction process’ stages (i.e. adoption, implementation, continuation) for physical activity interventions in primary healthcare [20]. These factors include financial feasibility of the intervention, intervention accessibility to the target group and time needed to deliver the intervention. However, different factors may affect implementation in different settings and to date there in no consensus regarding the importance of different factors.

Further consensus work has sought to identify frameworks and common implementation indicators to guide researchers in the design and evaluation of implementation and scale-up studies [21]. The use of theoretical frameworks for guiding implementation evaluations is recommended, as they provide a systematic approach to implementation using a common language to enable comparisons across studies and aid generalisability. [22, 23]. The most commonly referenced frameworks for implementation include the Framework for Effective Implementation [24] and the Consolidated Framework for Implementation Research (CFIR) [25]. Additionally, 25 implementation indicators were identified by the Delphi participants [20], with acceptability, adoption and adaptability of an intervention deemed most important for implementation success [21].

The potential of implementation frameworks such as the CFIR [25] to aid implementation of physical activity interventions has been further demonstrated by Czosnek et al. [26]. By exploring the literature and aligning it to the framework and its five domains (intervention characteristics, inner setting, outer setting, individual characteristics and processes of implementation), they provide recommendations for future research, including encouraging the use of research designs that combine assessment of intervention effectiveness and determinants of implementation (i.e. pragmatic trials with process evaluations). These constructs are summarized in Table 1 (adapted from [25]).

**Table 1.** Consolidated framework for implementation research

CFIR domains	Domain short description	Constructs	Example barriers and facilitators
Intervention characteristics	Attributes of an intervention that affect implementation	Intervention source (i.e. who/how was the intervention designed by) Evidence strength and quality Relative advantage Adaptability Trialability Complexity Design quality and packaging Cost	Barrier: costs associated with intervention Facilitator: evidence base provides credibility for intervention
Outer setting	Factors outside of the organization that affect implementation within the organization	Patient needs and resources Cosmopolitanism Peer pressure External policies and incentives	Barrier: cultural barriers Facilitator: community buy-in
Inner setting	Factors attributed to the host site or organization where implementation is planned that affects implementation	Structural characteristics Networks and communications Culture Implementation climate Readiness for implementation	Barrier: lack of support from leadership team Facilitator: dedicated time allowed for staff to deliver interventions
Characteristics of individuals	Attributes of individuals involved including patients, healthcare providers and leaders	Knowledge and beliefs about the intervention Self-efficacy Individual stage of change Individual identification with organization Other personal attributes	Barrier: lack of knowledge of intervention context Facilitator: High self-efficacy to deliver the intervention
Process	Stages involved in the implementation process	Planning Engaging Executing Reflecting and evaluating	Barrier: poor or missing implementation strategy Facilitator: engaging stakeholders throughout the entire intervention development and implementation

A recent qualitative systematic review [19] explored the barriers and facilitators to implementing physical activity interventions in a community setting for the general population of all age groups. Using the CFIR [25], 82 implementation factors (37 barriers and 45 facilitators) were identified and mapped to the five domains of the framework. It was noted that many of the barriers identified could have been reduced by the use of an implementation plan that included strategies for each stage of the process [19, 27]. Several frameworks have been developed to try and provide pragmatic advice, including a guide specific to physical activity, the PRACTical planning for Implementation and Scale-up (PRACTIS) guide [28]. By collating strategies to improve research–practice translation with constructs from implementation outcome, process and mechanistic models, including the CFIR [25], the guide aims to help researchers effectively plan the dissemination, implementation and scale-up of physical activity interventions into real-world settings.

## Implementation considerations: lessons from OA

In beginning to understand the implementation of physical activity interventions for people with IA, it is important to draw upon lessons learned in the context of related conditions such as OA.

Despite international guidelines that reflect the consistent body of evidence for physical activity as key in the management of OA, evidence suggests that care remains suboptimal [29–31] and physical activity is both underprescribed and underutilized [32–35]. A meta-analysis published in 2013 highlighted that across 21 studies of people with knee OA, only 13% of people met physical activity guidelines [36]. Suboptimal rates of exercise prescription were also reported by Steel et al. [37], who found that only 26% of eligible people with OA were prescribed an exercise program. Increasing attention has therefore been paid to the factors associated with the (lack of) uptake of this best evidence into clinical practice.

Factors influencing implementation are multifaceted and exist at the individual level [healthcare professionals (HCPs), patients and the public], organizational level (systems, processes, resources and culture) and a broader contextual level (policy) [38]. In addition, factors relating to the physical activity intervention itself may act as barriers and/or enablers to uptake. For example, demonstrating clinical and cost effectiveness may be of importance to stakeholders, however, flexibility of an intervention may enable stakeholder freedom to make contextually relevant adjustments to tailor the intervention to their individual staff, patient or organizational needs [39, 40]. Several studies have identified key factors that influence the implementation of guideline-based OA management programs and offer suggestions for optimizing the process.

Barriers to implementation at the individual level include the perception by both HCPs and patients that OA is a low-priority condition and the discordance between personal beliefs about the condition and recommended management [41, 42]. Personal and professional motivators, such as the desire to increase professional autonomy or to free up consultation time by way of onward referral, have also been shown to affect the implementation of key interventions, such as physical activity, for OA management [43]. Furthermore, a qualitative study informed by theory [the capability (C), opportunity (O), motivation (M) and behaviour (COM-B) model] [44] showed that the knowledge, skills and confidence of general practitioners in managing OA influenced the implementation of best practice in primary care [45].

At an organizational level, the need for implementation support such as resources, promotional and educational incentives for patients and HCPs to optimize the uptake of guideline-based OA management programs that include physical activity has been highlighted [44–47]. Individuals with credibility and influence within organizations, such as local champions or facilitators, can be instrumental in supporting ongoing implementation by providing infrastructure and resources to support the workload burden [47, 48]. In addition, engaging the whole practice and including sufficient time and opportunity for implementation planning and reflection were important strategies for optimizing implementation in a recent qualitative study [48]. A novel (and likely transferable) finding from this work was that public contributors and lay communities were not only recipients of healthcare innovations, but also powerful facilitators of implementation.

In considering system-related factors, the prioritization of other UK policy drivers, such as the Quality Outcomes Framework, which is associated with financial incentives, is a reported barrier to implementation [49]. Improved reimbursement models are suggested as a way to optimize the uptake of best practice [46], however, the identification of potential barriers early in the process may enable implementers to circumnavigate such barriers to optimize implementation.

The Enabling Self-management and Coping with Arthritic Pain through Exercise (ESCAPE-pain) program is one example of an evidence-based intervention for knee and hip OA that has been widely implemented across the UK [50]. Integrating self-management education with physical activity, the program [50] showed sustained clinical and cost effectiveness, yet the ongoing challenge and effort associated with implementation is well reported [47, 51]. Challenges included attitudes towards the evidence and evidence-based practice, persuading commissioners and providers to make small upfront investments to secure longer-term benefits and complex and fragmented funding and commissioning structures that created disconnects between commissioners and providers [40].

Factors that positively influenced implementation and scale-up of ESCAPE-pain included the strength and quality of evidence about the intervention, a quality suite of resources to facilitate implementation, local champions to drive sustained implementation and flexibility of the intervention whereby ESCAPE-pain was adopted across an expanding range of clinical and non-clinical community settings and delivered by a range of professionals utilizing a range of providers and funding arrangements [47].

While a 4-fold increase in the number of sites delivering ESCAPE-pain was seen over a 2 year period (from the start of the Academic Health Science Network national program in April 2018 to the publication of the full report detailing the evaluation of the program in March 2020) when ESCAPE-pain was delivered in 260 sites) [47], several ongoing challenges were identified. Poor links between the National Health Service (NHS) and non-NHS providers impeded implementation and sustaining the intervention required ongoing leadership and work [52], particularly by individuals whose role spanned operational and strategic functions. Evaluation of implementation was largely hampered by the lack of systems able to collect routine data and the burden of analysing data if/when collected. Conflicting drivers and agendas between academic and commissioning stakeholders illustrate the need for developing strong interstakeholder relationships and collaboration. Other suggested strategies for implementation include the use of financial measures to embed funding, contracting, the provision of free training for the implementation and delivery of the program, dedicated infrastructure and resources to support stakeholders with implementation and shared learning of barriers and facilitators across contexts.

## Exercise implementation in IA

Despite a wealth of trial evidence supporting physical activity as an intervention for IA [53], uptake is poor [9] and few studies have systematically studied interventions to support implementation. Patients report many potential reasons for not engaging in physical activity and a fear of exacerbating debilitating arthritis symptoms is frequently cited [54]. As with physical activity interventions for other conditions, understanding both the patient and organizational perspectives are key to successful implementation.

In the 1980s, the Arthritis Foundation in the USA developed and released two community-based programs for patients with arthritis (OA and IA): the Patients with Arthritis Can Exercise (PACE) program and a water-based program called the Arthritis Foundation Aquatic Program (AFAP) [55, 56]. PACE deliberately included the phrase ‘Can Exercise’ in its name and included an educational component to help address patient concerns that exercise would exacerbate arthritis symptoms or cause harm [56]. Although studies (some unpublished) point to beneficial outcomes for patients, program utilization (reach and participation) and quality control of program delivery were found to be low [55, 56].

Similar implementation issues occurred with other IA programmes. The Dutch Rheumatoid Arthritis Patients In Training (RAPIT) program examined the effectiveness and safety of a high-intensity exercise program in patients with RA, with the original trial [57] suggesting improvements in functional ability and emotional status with no detrimental effects on disease activity or radiographic progression. Wider implementation and evaluation was guided by the use of an implementation framework, with the Reach, Effectiveness, Adoption, Implementation and Maintenance framework used to establish the RAPIT program when expanding it across four regions in The Netherlands [58]. This wider rollout and adoption of the program revealed implementation challenges in terms of low patient participation levels; although two-thirds of participating rheumatologists referred patients to the program, only 2% of patients considered eligible actually registered to take part [58]. Concerns were also reported regarding program quality. Checklist audits assessing program materials, components and appropriateness of content, as well as attendance, found only 5 of 12 providers met quality criteria expectations [58].

Further implementation challenges may occur with dose and delivery of interventions when taken up into routine clinical practice. The Strengthening and Stretching for Rheumatoid Arthritis of the Hand (SARAH) trial showed that a tailored strengthening and stretching hand exercise program in addition to usual care led to improvements in hand function at 12 months and was considered to be both clinically and cost effective [59]. The original intervention [59] was six sessions of face-to-face contact with a physiotherapist or occupational therapist, but when implemented into a clinical setting the majority of therapists delivered only four sessions, often omitting behavioural strategies designed to support physical activity adherence [60]. However, the researchers anticipated potential implementation barriers and put strategies to overcome these in place. For example, in the SARAH program, face-to-face training for therapists was considered unfeasible when translating the program to nationwide delivery and the training was successfully moved to an online platform [60]. While intervention flexibility of delivery may be beneficial (by improving uptake), assessment of the impact of intervention changes is important, as when adaptations of interventions move away from the evidenced trial protocol, they may be less effective than the original trial. A recent systematic review [61] looked at how effective physical activity interventions were when they were scaled up from randomized controlled trials and suggested that most scaled-up interventions typically achieved <60% of their pre-scale effect size. This ‘scale-up penalty’ was often lower when the intervention was designed with the intent to be scaled up, highlighting the importance of considering implementation in trial design.

Other implementation barriers include cost and time. van den Berg et al. [62] undertook a trial of two internet-based physical activity interventions for people with RA and found that individually tailored supervision, exercise equipment and group contacts were more effective than an internet-based program alone. Scale-up implementation studies showed that although patients were eligible and interested in taking part and rheumatologists were willing to refer patients, the additional patient costs (as health insurance companies were only willing to partially reimburse the intervention) and the impact of ongoing physiotherapy were barriers to uptake [63].

Other studies also highlighted the impact of cost and time. Low participation rates were attributed to patients being unable to commit sufficient time to a PA program (e.g. RAPIT biweekly schedule over 12 months), financial cost (e.g. travel to program providers), lack of awareness of the program (e.g. PACE and AFAP) and psychological barriers such as a belief that physical activity provides limited benefits and low exercise self-efficacy. Variations in program delivery may be explained, in part, by organizational barriers such as issues in accessing equipment and limited capacity to deliver a program as intended [60]. However, these explanations for observed issues have been speculatively attributed (e.g. timing of exercise class may be inconvenient) or inferred from previous research (e.g. psychological barriers) and highlight inconsistencies in implementation evaluation processes.

Taken together with what is known about implementing physical activity programs in general, and in the specific contexts of OA and RA, will help future translation of physical activity programs for IA, although there remains a need for more systematic research to better understand implementation barriers and strategies that may address these [13, 61].

## Future recommendations for physical activity–based interventions for IA

To date, the majority of implementation research examining physical activity interventions for IA have only considered implementation factors at the implementation stage, despite evidence suggesting that scale up of interventions is more effective when implementation factors are considered early in the trial design stage. Physical activity programs are complex interventions containing several dimensions and behaviours. The Medical Research Council’s guidelines for evaluating complex interventions recognize the importance of incorporating theoretical frameworks to guide all stages of intervention development and refinement to enhance a program’s acceptability within the context it is intended to be delivered [13].

While it is encouraging that researchers are starting to use theoretical frameworks and implementation theory in intervention design [64–66], given the current inconsistencies in implementation assessment, it is difficult to establish which implementation strategies are most effective and why [21]. Adopting appropriate frameworks in the early stages of intervention development may help guide researchers in identifying and assessing key determinants for quality and consistency of implementation [18]. Furthermore, engaging with key professional and public stakeholders early may enable potential individual and organizational barriers to be addressed in the intervention design phase, thus supporting future successful implementation.

Given the research–practice gap, implementation is vital to aid translation of efficacious physical activity interventions from research contexts to real-world settings. Despite attempts to increase the knowledge base of factors influencing implementation, practical guidance for how clinicians and researchers can use this knowledge remains limited. Given the impact of physical inactivity on health and well-being for people with arthritis, further research to encourage uptake is urgently needed.

## Data Availability

No new data were generated or analysed in support of this research.
